# Mental health problems and associated school interpersonal relationships among adolescents in China: a cross-sectional study

**DOI:** 10.1186/s13034-020-00318-6

**Published:** 2020-03-30

**Authors:** Jiayu Li, Jing Li, Ruixia Jia, Yingquan Wang, Sheng Qian, Yong Xu

**Affiliations:** 1 Department of Child and Adolescent Health, School of Public Health, Medical College of Soochow University, No. 199 Ren Ai Road, Suzhou, 215123 Jiangsu People’s Republic of China; 2 Department of Social Medicine, Jiangsu Key Laboratory of Preventive and Translational Medicine for Geriatric Diseases, School of Public Health, Soochow University, Suzhou, 215123 Jiangsu People’s Republic of China

**Keywords:** Chinese adolescents, Mental health problems, Teacher–student relationship, Peer relationship, Symptom Checklist-90 (SCL-90)

## Abstract

**Background:**

During adolescence, middle school students facing psychophysical changes are vulnerable to psychological problems. The present study aimed to investigate mental health status and associated school interpersonal relationships among adolescents in China, which may help to inform effective prevention strategies to reduce the prevalence of mental health problems.

**Methods:**

In the cross-sectional study, a total of 10,131 middle school students were selected from three cities in eastern China by stratified random sampling. The Symptom Checklist-90 (SCL-90), Teacher–Student Relationship Questionnaire (TSRQ) and Peer Relationship Scale (PRS) were used to evaluate psychological symptoms, the quality of relationship with teachers and the quality of relationship with peers, respectively. Multivariable logistic regression analysis was conducted to explore the association between school interpersonal relationships and mental health problems in adolescents.

**Results:**

36% of the middle school students reported positive in mental health problems assessed by the SCL-90. The most prevalent dimensional symptom was obsessive–compulsive (43.3%). The risk of all types of psychological symptoms was significantly associated with school interpersonal relationships. Moreover, a higher risk of mental health problems was associated with poorer school interpersonal relationships.

**Conclusions:**

Mental health problems were prevalent among adolescents and highly associated with school interpersonal relationships. Our findings underscore the pressing need for school administrators to make efforts to improve school interpersonal relationships among adolescents.

## Background

Adolescence has been characterized by heightened emotional reactions and perceived to be a peak period for a substantial proportion of mental health problems [1]. Almost 50% of all mental health problems, including depression, anxiety and aggressive behavior, seem to start by the age of 14 years [2]. Mental health problems are a pressing public health issue among adolescents with the prevalence rates of about 10–20% worldwide [3]. Neuropsychiatric and substance abuse disorders are the leading causes of disease burden in adolescents in all regions [4]. Poor mental health among adolescents is associated with serious long-lasting consequences, such as lower educational attainment, increased health-care costs, substance abuse, violence, self-harm and even suicide, which are likely to persist into adulthood [5, 6]. In addition, 9-year compulsory education has been implemented in China’s education system. Most of students enter senior secondary schools after they received primary and junior secondary education. In order to get well prepared for senior secondary school entrance examination and college entrance examination, students face tremendous pressure from study and competition [7, 8]. A competitive education system in traditional Chinese culture has been linked with the high incidence of anxiety among adolescents [9, 10]. According to the Chinese National Bureau of Statistics, there are about 1.4 billion people in China, among which 235 million are people under the age of 15. Given that most mental disorders are likely to originate in childhood and have devastating impacts [11, 12], there is an urgent need to strengthen early identification and prevention for the vulnerable population.

Previous studies have reported that teacher–student relationship and peer relationship are linked to the psychological well-being of adolescents [13, 14]. For adolescents, a positive relationship with their teachers and classmates seems to diminish the negative life experience and favour the psychological development simultaneously [15, 16]. Published studies demonstrated that the social support provided by teachers may be related to the mental health of students [17]. Conversely, students suffering from internalizing problems (i.e., anxiety and depression) and externalizing problems (i.e., aggressive and oppositional behavior) tend to have a negative relationship with teachers [18]. In addition, students who have a low-quality peer relationship reported a higher risk of psychological symptoms, social obstacles and academic failure [19, 20]. Specifically, middle school students in puberty confronted enormous challenges which may have effects on their mental and physical health for a prolonged period. Previous studies documented that the influence of peers is most pronounced during adolescence [21]. It is worth noting that peers and school environment gradually play an important role in puberty, meanwhile, the impact of the family tend to fade [22, 23]. Therefore, it is of utmost importance to explore the relation between mental health problems and school interpersonal relationships among adolescents. Concerning this important issue, available studies are generally carried out in Western countries. There is a paucity of studies focusing on the association between school interpersonal relationships and mental health problems among adolescents in China. Having a better understanding of psychological symptoms and associated risk factors in school among this vulnerable population can arouse more attention from all circles in society, which is conducive to the prevention of mental health problems in the world’s most populous country.

To date, previous studies of psychological symptoms among adolescents have been conducted within the narrow confines of geographic borders in China. In this study, we aimed to comprehensively assess the prevalence of mental health problems with a large sample and examine the relation between mental health problems and basic interpersonal relationships in school among adolescents. Inspired by findings of previous studies [5, 20, 24], we hypothesized that mental health problems are a pressing public health issue among adolescents in China. In addition, mental health problems among adolescents in China are positively linked to poor teacher–student, and peer relationship.

## Materials and methods

### Participants and procedure

We conducted this cross-sectional study in Hangzhou, Suzhou and Hefei. Middle school students were selected by a two-stage cluster random sampling method. In the first stage, 6 junior secondary schools (aged 13–15 years) and 6 senior secondary schools (aged 16–18 years) were randomly selected in each city, resulting in a total of 36 schools. In the second stage, 2 classes from each grade at each school were randomly selected. Finally, a total of 10,184 students in 216 classes were invited to complete the survey in September through December 2018. The survey was spread out over the school year and avoided during the school examination period. Before the study commenced, students were informed of the purpose of the study, and written informed consents were obtained from students as well as one of their parents or legal guardians. To avoid information bias, students were asked to complete survey questionnaires anonymously in the absence of teachers during class time. All the data was considered as confidential to protect the privacy of study participants. In the end, 10,131 valid surveys were retained after some questionnaires with missing items were excluded. Previous studies have shown that 4.0–36.3% of adolescents experience psychological problems in China [25, 26]. To ensure statistical efficacy, the lowest prevalence rate (4.0%) was used to estimate the minimum required sample size. The sample size was calculated by the following formula ${\text{n}} = \left\{ {57.3{\text{Z}}_{\alpha /2} /{\text{arcsin}} \left[ {\varepsilon {\text{P/}}\sqrt {{\text{P}}\left( {1 - {\text{P}}} \right)} } \right]} \right\}^{2}$. In the present study, p = 0.04 (representing the prevalence of mental health problems), ɛ = 0.1 (representing permitted minimum error), α = 0.05 (representing I type error, Z_ɑ/2_ = 1.96 accordingly), and a 10% non-respondent rate or missing was considered, the sample size was estimated to be 10,097 participants. Finally, a total of 10,184 students were invited to participate in the survey because of some larger classes.

This study was reviewed and approved by the ethics committee of the Health Development Research Center of Soochow University in Suzhou, Jiangsu, China. In accordance with Declaration of Helsinki, we obtained written informed consents from all students and one of their parents or legal guardians before being surveyed.

### Measures

#### Confounding factors

To gain a better understanding of potential confounding factors, we collected social demographic characteristics including age, sex, sibling status, household and family economic status. In this study, students rated their family economic conditions with the option of good, middle or poor, since middle school students have the capacity to rate their perceived family economic status. While in class, research assistants distributed the questionnaires to students in the absence of teachers.

#### Mental health problems

Psychological status was measured by the SCL-90, a self-report mental health questionnaire designed to screen for a broad range of psychological problems [27]. The SCL-90 consisted of 90 items, including the following nine psychiatric symptom factors: somatization (SOM), obsessive–compulsive (O–C), interpersonal sensitivity (I-S), depression (DEP), anxiety (ANX), hostility (HOS), phobic anxiety (PHOB), paranoid ideation (PAR), psychoticism (PSY). Each item of the SCL-90 was rated on a five-point likert scale ranging from 1 (not at all) to 5 (extremely). The higher the scores of SCL-90 (ranged between 90 and 450) were associated with the more serious of the psychological problems during the last week. Based on the application of SCL-90 in the Chinese population, the participant was considered as having mental health problems in the screening if the total score is > 160 points [28]. The severity of the specific psychological problem was reflected by each subscale score. For instance, individuals with a standardized subscale score (the sum of all the subscale scores divided by the number of items) ≥ 3 were considered as having specific moderate to severe psychological problem, while individuals with a standardized subscale score between 2 and 3 were considered as having specific mild psychological problem [29]. According to previous studies, the SCL-90 was demonstrated to have good psychometric properties with satisfactory reliability and validity [30, 31]. The Cronbach’s alpha coefficient for SCL-90 in this study was 0.91.

### Exposure variables

#### Teacher–student relationship

The teacher–student relationship was evaluated by the TSRQ, a self-report instrument revised by Qu et al. [32]. The questionnaire comprises 23 items and has been wildly used among adolescents in China [33]. It constitutes four primary dimensions: intimacy, satisfaction, support and conflict. Each item of the questionnaire is rated by the student on a five-point scale ranging from 1 (completely disagree) to 5 (completely agree). In the current study, the total score of each dimension ≥ 75th percentile of the score distribution was classified as ‘good’, greater than 25th percentile but less than 75th percentile as ‘middle’ and < 25th percentile as ‘poor’, while the dimension of conflict works contrariwise. In this study, the Cronbach’s alpha for each dimension of TSRQ ranged from 0.71 to 0.87.

#### Peer relationship

We used the PRS developed by Wei et al. to measure the quality of peer relationship [24]. The PRS consisted of 20 items and was well validated in previous study [34]. The items are divided into three dimensions: social-emotional, communicative interaction and interpersonal harmony. Each item of the questionnaire is rated on a five-point Likert scale with responses ranging from 1 (completely disagree) to 5 (completely agree). In this study, the total score of each dimension ≥ 75th percentile of the score distribution was classified as ‘good’, greater than 25th percentile but less than 75th percentile as ‘middle’ and < 25th percentile as ‘poor’. The Cronbach’s alpha for each dimension of PRS ranged between 0.70 and 0.83.

### Data analysis

All statistical analyses were performed with the Statistical Package for the Social Sciences (SPSS Inc., Chicago, IL, USA) version 20.0. Descriptive statistics were performed on sociodemographic characteristics and were displayed properly in frequencies and proportions. Univariable analyses were conducted to identify any associations between sociodemographic characteristics, teacher–student relationship and peer relationship and mental health problems. The dependent variable were mental health problems (SCL-90 > 160) or specific psychological problems (the sum of all the subscale scores divided by the number of items ≥ 2). Multivariable logistic regression analysis was constructed to explore the impact of teacher-student relationship and peer relationship on the risk of mental health problems (SCL-90 > 160). Model 1 was adjusted for all dimensions of TSRQ and PRS, and model 2 was adjusted for variables in model 1 and gender, age, sibling status, household and family economic status. The results were shown in the form of odds ratios (OR) and 95% confidence interval (CI). All statistical tests were two-sided, with p value < 0.05 was regarded as statistically significant.

## Results

### Mental health problems and sociodemographic characteristics of the participants

A total of 10,131 middle school students with valid data were included in the present study, comprising 4881 boys (48.2%) and 5250 girls (51.8%). The age of the participants ranged from 13 to 18 years (mean age = 15.05, SD = 1.66). The sociodemographic characteristics of the participants were summarized in Table 1. Overall, 5584 (55.1%) students were the only child status, 4547 (44.9%) students were not the only child status in their family. In addition, a total of 5353 (52.8%) students came from rural areas, and 4778 (47.2%) students came from urban areas. In terms of family economic status, 1514 (14.9%) students reported that their family economic status was good, 7111 (70.2%) students reported that their family economic status was modest, while 1506 (14.9%) students reported that their family economic status was poor. The risk of mental health problems was significantly higher among girls, older age groups, having siblings, rural household and poor family economic status groups than in boys, younger age groups, one-child status, urban household and good family economic status groups, without adjusting for other variables.

**Table 1:** Prevalence of mental health problems in middle school students by demographic factors

Demographic characteristics	N	Prevalence (%)	p-value	OR (95% CI)
Gender				
Female	5250	37.3	–	1
Male	4881	34.7	0.006	0.893 (0.823–0.968)
Age			< 0.001^a^	
13	2308	23.6	–	1
14	2251	38.2	< 0.001	2.000 (1.759–2.274)
15	1663	38.9	< 0.001	2.060 (1.795–2.364)
16	1631	41.3	< 0.001	2.278 (1.985–2.614)
17	1120	37.5	< 0.001	1.941 (1.663–2.265)
18	1158	43.4	< 0.001	2.484 (2.137–2.888)
Being only child				
No	4547	38.0	–	1
Yes	5584	34.4	< 0.001	0.854 (0.787–0.927)
Household registration				
Rural	5353	38.3	–	1
Urban	4778	33.4	< 0.001	0.809 (0.746–0.878)
Family economic status				
Good	1514	33.4	–	1
Middle	7111	34.1	0.597	1.032 (0.918–1.161)
Poor	1506	47.5	< 0.001	1.805 (1.559–2.092)

*SOM* somatization, *O–C* obsessive–compulsive, *I-S* interpersonal sensitivity, *DEP* depression, *ANX* anxiety, *HOS* hostility, *PHOB* phobic anxiety, *PAR* paranoid ideation, *PSY* psychoticism

OR, OR after univariable logistic regression

^a^p value for trend

### The prevalence of mental health problems

As shown in Table 2, 64.0% of students were within normal range with SCL-90 score under 160. With regard to the dimensional symptoms, 15.7% of students reported mild and 5.3% of students reported moderate to severe level of somatization symptoms. A total of 30.6% of students reported mild and 12.7% of students reported moderate to severe level of obsessive–compulsive symptoms. A total of 25.6% of students reported mild and 10.1% of students reported moderate to severe level of interpersonal sensitivity symptoms. A total of 20.7% of students reported mild and 8.2% of students reported moderate to severe level of depression symptoms. A total of 20.9% of students reported mild and 9.0% of students reported moderate to severe level of anxiety symptoms. A total of 21.7% of students reported mild and 10.4% of students reported moderate to severe level of hostility symptoms. A total of 18.3% of students reported mild and 8.5% of students reported moderate to severe level of phobic anxiety symptoms. A total of 22.6% of students reported mild and 8.7% of students reported moderate to severe level of paranoid ideation symptoms. A total of 18.0% of students reported mild and 7.0% of students reported moderate to severe level of psychoticism symptoms.

**Table 2:** Prevalence of different levels of mental health problems, n (%)

Dimensions	Normal	Mild	Moderate to severe
SOM	8004 (79.0)	1586 (15.7)	541 (5.3)
O–C	5748 (56.7)	3093 (30.6)	1290 (12.7)
I-S	6514 (64.3)	2598 (25.6)	1019 (10.1)
DEP	7205 (71.1)	2099 (20.7)	827 (8.2)
ANX	7099 (70.1)	2117 (20.9)	915 (9.0)
HOS	6875 (67.9)	2200 (21.7)	1056 (10.4)
PHOB	7411 (73.2)	1859 (18.3)	861 (8.5)
PAR	6957 (68.7)	2286 (22.6)	888 (8.7)
PSY	7607 (75.0)	1822 (18.0)	702 (7.0)
Total score	6482 (64.0)	–	–

*SOM* somatization, *O–C* obsessive–compulsive, *I-S* interpersonal sensitivity, *DEP* depression, *ANX* anxiety, *HOS* hostility, *PHOB* phobic anxiety, *PAR* paranoid ideation, *PSY* psychoticism

### The association between school interpersonal relationships and mental health problems

As shown in Table 3, mental health problems were significantly associated with all dimensions of the TSRQ and PRS, without adjustment for other variables. An increased odds ratio of each psychological subscale was associated with the level and all dimensions of teacher–student relationship and peer relationship among students. Moreover, compared with students who reported positive teacher–student relationship and peer relationship, the risk of mental health problems were significantly higher for students who reported negative teacher-student relationship and peer relationship, indicating that negative school interpersonal relationships tend to be associated with a higher risk of mental health problems. The mean scores and SDs of TSRQ and PRS were shown in Additional file 1: Table S1.

**Table 3:** Univariable logistic regression analysis of school interpersonal relationships associated with psychological problems, OR (95% CI)

Variables	SOM	O–C	I-S	DEP	ANX	HOS	PHOB	PAR	PSY	Total score
Teacher–student relationship										
Intimacy										
Good (2357)	1	1	1	1	1	1	1	1	1	1
Middle (5203)	1.306 (1.152–1.482)	1.661 (1.499–1.840)	1.593 (1.430–1.776)	1.564 (1.392–1.756)	1.453 (1.298–1.628)	1.437 (1.287–1.604)	1.330 (1.184–1.493)	1.551 (1.386–1.736)	1.508 (1.334–1.705)	1.616 (1.450–1.801)
Poor (2571)	1.523 (1.324–1.753)	2.375 (2.114–2.667)	2.363 (2.094–2.666)	2.130 (1.874–2.421)	1.999 (1.763–2.266)	1.999 (1.768–2.259)	1.735 (1.525–1.973)	2.187 (1.931–2.478)	2.146 (1.876–2.454)	2.440 (2.162–2.752)
Satisfaction										
Good (2301)	1	1	1	1	1	1	1	1	1	1
Middle (4171)	1.199 (1.050–1.369)	1.300 (1.170–1.445)	1.365 (1.220–1.526)	1.318 (1.169–1.487)	1.244 (1.107–1.398)	1.151 (1.027–1.289)	1.185 (1.050–1.336)	1.251 (1.114–1.405)	1.307 (1.151–1.486)	1.365 (1.220–1.526)
Poor (3659)	1.602 (1.404–1.828)	1.791 (1.609–1.993)	1.982 (1.771–2.219)	1.956 (1.735–2.204)	1.693 (1.506–1.903)	1.667 (1.488–1.867)	1.595 (1.414–1.800)	1.898 (1.690–2.131)	2.026 (1.786–2.300)	2.054 (1.836–2.299)
Support										
Good (1785)	1	1	1	1	1	1	1	1	1	1
Middle (5230)	1.611 (1.385–1.875)	1.845 (1.644–2.070)	1.819 (1.607–2.059)	1.883 (1.643–2.158)	1.768 (1.550–2.016)	1.632 (1.439–1.851)	1.516 (1.326–1.732)	1.903 (1.666–2.172)	1.755 (1.517–2.030)	1.901 (1.678–2.153)
Poor (3116)	2.345 (2.004–2.744)	2.265 (2.003–2.562)	2.599 (2.280–2.963)	2.689 (2.332–3.101)	2.378 (2.071–2.731)	2.216 (1.940–2.531)	2.111 (1.835–2.429)	2.869 (2.497–3.296)	2.882 (2.480–3.350)	2.730 (2.393–3.113)
Conflict										
Low (2908)	1	1	1	1	1	1	1	1	1	1
Middle (4991)	2.113 (1.852–2.412)	2.129 (1.932–2.346)	2.174 (1.959–2.412)	2.378 (2.115–2.673)	2.318 (2.070–2.597)	2.271 (2.034–2.535)	1.840 (1.641–2.062)	2.584 (2.305–2.896)	2.642 (2.324–3.004)	2.370 (2.133–2.633)
High (2232)	3.620 (3.133–4.183)	2.537 (2.262–2.846)	2.991 (2.652–3.374)	4.023 (3.530–4.585)	3.503 (3.082–3.983)	3.521 (3.107–3.990)	2.757 (2.424–3.136)	4.078 (3.586–4.637)	4.536 (3.940–5.221)	3.359 (2.975–3.793)
Peer relationship										
Social-emotional										
Good (2423)	1	1	1	1	1	1	1	1	1	1
Middle (4648)	1.395 (1.229–1.584)	1.418 (1.281–1.569)	1.635 (1.467–1.822)	1.516 (1.351–1.702)	1.485 (1.327–1.663)	1.699 (1.518–1.901)	1.386 (1.234–1.558)	1.514 (1.353–1.693)	1.512 (1.338–1.708)	1.582 (1.420–1.761)
Poor (3060)	1.451 (1.267–1.663)	1.696 (1.521–1.892)	2.024 (1.803–2.272)	1.841 (1.629–2.081)	1.694 (1.502–1.911)	2.004 (1.778–2.259)	1.581 (1.396–1.789)	1.844 (1.637–2.077)	1.805 (1.587–2.054)	1.945 (1.734–2.182)
Communicative interaction										
Good (2107)	1	1	1	1	1	1	1	1	1	1
Middle (5335)	1.395 (1.221–1.595)	1.555 (1.399–1.729)	1.875 (1.671–2.105)	1.626 (1.438–1.838)	1.747 (1.547–1.972)	1.534 (1.367–1.721)	1.408 (1.248–1.589)	1.621 (1.440–1.826)	1.705 (1.495–1.945)	1.793 (1.600–2.009)
Poor (2689)	1.723 (1.490–1.993)	2.092 (1.859–2.354)	2.659 (2.343–3.017)	2.353 (2.061–2.688)	2.223 (1.948–2.537)	1.847 (1.627–2.097)	1.605 (1.405–1.834)	2.385 (2.096–2.715)	2.414 (2.096–2.781)	2.338 (2.063–2.649)
Interpersonal harmony										
Good (2417)	1	1	1	1	1	1	1	1	1	1
Middle (4712)	1.555 (1.362–1.774)	1.608 (1.451–1.782)	1.890 (1.689–2.115)	1.842 (1.630–2.081)	1.813 (1.612–2.040)	1.618 (1.444–1.812)	1.621 (1.436–1.829)	1.669 (1.486–1.874)	1.703 (1.498–1.935)	1.893 (1.693–2.117)
Poor (3002)	1.991 (1.732–2.288)	2.199 (1.967–2.457)	3.021 (2.681–3.404)	2.906 (2.558–3.302)	2.359 (2.082–2.672)	2.327 (2.064–2.624)	2.151 (1.894–2.444)	2.562 (2.268–2.895)	2.641 (2.312–3.016)	2.788 (2.477–3.140)

*SOM* somatization, *O–C* obsessive–compulsive, *I-S* interpersonal sensitivity, *DEP* depression, *ANX* anxiety, *HOS* hostility, *PHOB* phobic anxiety, *PAR* paranoid ideation, *PSY* psychoticism

All p values are significant at p < 0.05

### Multivariable logistic regression analysis on mental health problems with school interpersonal relationships

The results of the multivariable logistic regression analysis (Table 4) showed that each dimension of TSRQ and PRS was significantly associated with mental health problems. In Model 1, support and social-emotional were not associated with mental health problems. However, there was a tendency for poorer school interpersonal relationships to be associated with a higher risk of mental health problems after further adjustment for sex, age, sibling status, household and family economic status simultaneously in Model 2. Clearly, of all dimensions of TSRQ and PRS, conflict in TSRQ had the strongest association with mental health problems, followed by interpersonal harmony in PRS and support in TSRQ.

**Table 4:** Multivariable logistic regression analysis for mental health problems

Variables	Model 1		Model 2	
	p	OR (95% CI)	p	OR (95% CI)
Teacher–student relationship				
Intimacy				
Good	–	1	–	1
Middle	0.096	1.112 (0.981–1.259)	< 0.001	1.490 (1.334–1.664)
Poor	< 0.001	1.414 (1.219–1.639)	< 0.001	2.175 (1.922–2.460)
Satisfaction				
Good	–	1	–	1
Middle	0.429	1.051 (0.929–1.190)	< 0.001	1.299 (1.159–1.456)
Poor	0.004	1.225 (1.067–1.405)	< 0.001	1.919 (1.711–2.152)
Support				
Good	–	1	–	1
Middle	0.064	1.142 (0.992–1.314)	< 0.001	1.680 (1.479–1.908)
Poor	0.139	1.131 (0.961–1.331)	< 0.001	2.395 (2.093–2.740)
Conflict				
Low	–	1	–	1
Middle	< 0.001	1.892 (1.692–2.116)	< 0.001	2.254 (2.024–2.510)
High	< 0.001	2.757 (2.415–3.148)	< 0.001	3.246 (2.863–3.680)
Peer relationship				
Social-emotional				
Good	–	1	–	1
Middle	0.517	0.956 (0.835–1.095)	< 0.001	1.493 (1.338–1.666)
Poor	0.065	0.859 (0.731–1.009)	< 0.001	1.841 (1.636–2.071)
Communicative interaction				
Good	–	1	–	1
Middle	0.005	1.227 (1.063–1.415)	< 0.001	1.682 (1.498–1.889)
Poor	0.059	1.185 (0.994–1.414)	< 0.001	2.127 (1.871–2.418)
Interpersonal harmony				
Good	–	1	–	1
Middle	< 0.001	1.462 (1.268–1.687)	< 0.001	1.788 (1.596–2.004)
Poor	< 0.001	1.926 (1.618–2.293)	< 0.001	2.644 (2.339–2.988)

Model 1 was adjusted for all dimensions of TSRQ and PRS

Model 2 was adjusted for variables in model 1 and gender, age, sibling status, household and family economic status

## Discussion

In the present study, we found that mental health problems were a serious public health issue among Chinese adolescents, with 36.0% of students reported positive mental health problems based on the total score of SCL-90. The most common dimensional symptoms were obsessive–compulsive (43.3%), followed by interpersonal sensitivity (35.7%) and hostility (32.1%). Moreover, this study revealed that the positive associations between school interpersonal relationships and mental health problems, which was independent of potential confounding factors, including sex, age, sibling status, household and family economic status. The risk of mental health problems was increased progressively with poorer teacher–student relationship and peer relationship.

Our study showed a higher prevalence of obsessive–compulsive symptoms than some other foreign studies [35, 36]. For example, Ran et al. reported that the prevalence of obsessive–compulsive symptoms was 38.2% in the United States among community youth, which including 7054 representative participants. In contrast with similar studies conducted in China, our data showed a higher prevalence of obsessive–compulsive symptoms than that found by Yao et al. [37], who found a prevalence of 35.9% in southeast China, and lower prevalence than that reported by Wang et al. [38], who found a prevalence of 66.0% in north China. These discrepancies may be partly explained by the unbalanced economic development between different regions. Nevertheless, in light of the varied screening methods and different populations, the comparison of the prevalence of psychological symptoms among relevant reports should be interpreted with caution. Our study indicated that obsessive–compulsive symptoms were prevalent among adolescents. This could partly be explained by increasing negative life events and enormous academic pressure [39, 40].

In addition, univariable analysis showed that mental health problems were significantly associated with gender, age, sibling status, household and family economic status. The finding of our study is consistent with the view that girls have a higher risk of experiencing mental health problems than boys [5, 41, 42]. The older age groups reported significantly higher risk of psychological symptoms, which was in line with previous surveys in England [43]. Our study showed that students from low-income families were more prone to be distressed by psychological symptoms, which was in agreement with previous researches [44–47]. Compared with students from urban areas, students from rural areas were more prone to mental disorders, which were consistent with previous studies [48–50]. In accordance with previous findings, the present study indicated that students with siblings were more likely to suffer from mental health problems [51, 52].

Our findings suggest that poorer teacher–student relationship and peer relationship might have negative effects on mental health problems among adolescents after adjustment for potential confounders. Longobardi et al. reported that positive teacher–student relationship, such as higher levels of closeness and lower levels of conflict, was a protective factor not only for the cognitive development but also for the emotional function of students [53]. Furthermore, published studies demonstrated that school satisfaction and social support from teacher had an important role in the development of mental and physical well-being among adolescents [54]. Therefore, schools should attach more importance to develop the communication skills between teachers and students. Further studies will be needed to focus on the association between teacher–student relationship and mental health problems among adolescents. This result is consistent with some previous studies, which indicated that peer problems can affect the emotional well-being of adolescents [55, 56]. It has been shown that the psychological and behavioral characteristics of adolescents, such as externalizing and internalizing problems, were affected by their peers [57–59]. So it is essential for us to underline the significance of peer influence to reduce the prevalence of mental health problems among adolescents. Moreover, in the school setting, adolescents spend a lot of time getting along with teachers and peers who replace parents as significant others. A positive relationship with teachers and peers can develop a harmonious environment and contribute to students’ well-being [16]. In the meanwhile, difficulties in social situations exacerbate the mental health problems, and in turn poor mental health deteriorates school interpersonal relationships. Therefore, harmonious school interpersonal relationships are essential for the overall development of adolescents.

The present study is one of the few studies exploring the association between school interpersonal relationships and mental health problems of adolescents. We had a large sample size and a multidimensional scale which evaluates a board range of psychological problems simultaneously. An ample amount of details provided by the SCL-90 may have implications for some pointed intervention measures to improve the mental health of middle school students. However, our study also has some limitations. First, it must be stressed that the accurate causality between predicative variable and dependent variable cannot be determined because of the cross sectional study design. Second, all information was based on self-report data, which might result in reporting bias. Specifically, though adolescents have the capacity to rate their perceived family economic status to some extent, the accuracy of the self-reported family economic status without proxy measures cannot be guaranteed. Third, the results may not be representative of all adolescents in China, because this study only collected data from middle school students. In addition, the same numbers of schools were selected from three cities and weight adjustment of the estimates was not conducted, hence, the representativeness of the result may be limited. Finally, it should be noted that we did not take into account other risk factors, such as parent–child relationship, parental education and occupation, family history of psychotic disorders, living environment and living habits, future studies with full consideration are needed to clarify this. Moreover, some academic performance variables were not considered, such as academic pressure and academic achievement [60, 61], which may be associated with mental health problems among school-going adolescents, thus potential residual confounding could not be excluded in this study. Given these aforementioned limitations, further cohort researches with more representative samples and sophisticated design need to be carried out.

## Conclusions

In conclusion, the current study shows that mental health problems are prevalent among adolescents, especially obsessive–compulsive symptoms. Regression analysis reveals that poorer interpersonal relationships in school were associated with higher risk of mental health problems among adolescents, which suggests the pressing need for school educators to make efforts to improve school interpersonal relationships. Our findings have an important implication for practitioners to understand how school interpersonal relationships are linked to mental health problems among adolescents, which can provide insights for potential interventions. To improve school interpersonal relationships of adolescents, policymakers and teachers should integrate mental health education into various educational activities and build a harmonious school culture. Schools should also provide students with guidance for maintaining a healthy and positive mental state. Moreover, further exploration focused on the impact of school interpersonal relationships is desperately needed in China.

## Supplementary information

**Additional file 1: Table S1.** The mean scores and SDs of TSRQ and PRS.

## Data Availability

The datasets used during the current study are available from the corresponding author on reasonable request.

## Abbreviations

SCL-90: The Symptom Checklist-90
TSRQ: Teacher–Student Relationship Questionnaire
PRS: Peer Relationship Scale
SOM: Somatization
O–C: Obsessive–compulsive
I-S: Interpersonal sensitivity
DEP: Depression
ANX: Anxiety
HOS: Hostility
PHOB: Phobic anxiety
PAR: Paranoid ideation
PSY: Psychoticism
OR: Odds ratios
CI: Confidence interval
